# Better Together?: A Systematic Review of the Effectiveness of HIV Prevention Interventions that Build or Strengthen Social Ties

**DOI:** 10.1007/s10461-025-04745-4

**Published:** 2025-05-15

**Authors:** Virginia A. Fonner, Ping Teresa Yeh, Caitlin E. Kennedy, Kevin R. O’Reilly, Michael D. Sweat

**Affiliations:** 1 Global Programs and Science, FHI 360 359 Blackwell St #200, Durham, NC 27701 USA; 2 Social and Behavioral Interventions Program, Department of International Health, Johns Hopkins Bloomberg School of Public Health, Baltimore, MD USA; 3 Division of Global and Community Health, Department of Psychiatry, Medical University of South Carolina, Charleston, SC USA

**Keywords:** HIV prevention, Social relationships, Social capital, Community mobilization, Human capital, Community engagement, Peer support

## Abstract

Although social relationships impact health and social dynamics play a key role in shaping HIV vulnerability, results from intentional efforts to build or strengthen social relationships have not been synthesized to understand if and how such interventions work to prevent HIV in low- and middle-income countries (LMICs). We conducted a systematic review of HIV prevention interventions implemented in LMICs, published between 2010 and 2022, that used pre/post or multi-arm methods to evaluate interventions that focused on building/strengthening social relationships to impact HIV-related outcomes. We searched PubMed, CINAHL, EMBASE, Sociological Abstracts, and PsycInfo on August 16, 2022, complemented by hand-searching and secondary reference searching. We used a standardized form for data abstraction and assessed risk of bias using the Evidence Project tool. Results were synthesized narratively, and studies were classified in an emergent typology based on the function of social tie building within the intervention. Fifty-one articles presenting results from 28 studies met the inclusion criteria and were included. Within these studies, we identified five types of social tie interventions, including community-wide social mobilization (“collaboration”, *n* = 3), formation of collectives to address both upstream and downstream health-related factors (“collectivization”, *n* = 13), forming or strengthening groups to enhance peer support and build skills (“clubs”, *n* = 4), expanding personal networks among individuals (“companionship”, *n* = 2), and strengthening ties between heterogeneous groups/non-peers (“connections”, *n* = 2). Four studies addressed two or more types of social ties strengthening and were classified as “cross-cutting.” Across these categories, most studies found that interventions were associated with some positive health-related changes, such as reduced HIV incidence, increased condom use, and increased health service utilization. However, some interventions fell short of their stated goals, especially those striving to impact upstream social and structural factors. Overall, results suggest that social ties can be intentionally altered to effect change; however, disparate contexts and implementation dynamics likely contributed to variation seen across outcomes and impact. Inconsistent measurement of social ties and use of theory made it challenging to determine whether interventions were explicitly trying to alter ties, and if so, to what extent tie building/strengthening impacted intervention effectiveness. To continue advancing our understanding of social tie interventions, more efforts are needed to operationalize theory, measure social tie constructs, describe intervention context and implementation outcomes, and apply innovative study designs.

## Introduction

Social Relationships Impact Health

Beginning in part with the seminal work of Berkman and Styme linking social relationships with mortality risk [1], this conclusion has been widely supported both empirically and theoretically. Development of theories in fields such as sociology (e.g., Durkheim [2]) and psychology (e.g., Bowlby [3]) have helped explain how social interactions are critical for developing our sense of belonging in the world. Empirically, many studies have linked benefits of social connections, including social support, with a host of health-related behaviors and outcomes, such as prevention of cardiovascular disease [4], self-management of diabetes mellitus [5], and mental health [6]. However, science is still grappling with understanding mechanisms through which social ties impact health, and whether these pathways can be deliberately acted upon to effect change. In this review, we focus on how interventions have deliberately sought to build or strengthen social ties within the field of HIV prevention, specifically in low–and middle–income countries, which often have the highest HIV burden—and whether such interventions were able to achieve measurable health impacts.

The importance of social ties within HIV prevention is rooted in the early days of the HIV epidemic in high-income countries, like the United States, where sexual and gender minority communities—disproportionately impacted by HIV—faced exceptional stigma, resulting in adverse consequences such as denial of health service and lack of adequate prevention resources [7, 8]. In response, various communities and activists came together to raise awareness about HIV, provide critical information and community support, and advocate for change using a rights-based approach [8, 9]. These pioneering initiatives were organic, grassroots movements born from necessity. In more recent years, interventions—often created in partnership with communities in which they are implemented—have sought to engineer similar social mobilization campaigns among marginalized populations impacted by HIV, such as sex workers in India [10, 11], in part through strengthening ties among sex workers to foster collective action (e.g., demanding access to quality healthcare services and advocating for social change) [12]. These are examples of social ties impacting health through altering upstream social and structural factors and are often geared to addressing health inequities. Relevant theories that encompass this type of social tie building include social capital and community mobilization, although definitional ambiguities and operationalization challenges related to their application have been noted [13–16].

Peer-based interventions related to HIV prevention are relatively common and have shown effectiveness with respect to changing sexual risk behaviors and HIV-related knowledge [17, 18], but these interventions typically focus on using existing peer groups as conduits for disseminating information or altering group norms and are not typically focused on building or strengthening ties. Existing theories and concepts, such as social support and social integration, suggest that strengthening or forging new ties within existing peer groups could lead to improved outcomes [19]. Social support typically refers to resources, including material, psychological, or emotional, provided to an individual or group to alleviate stress and provide aid [20, 21]. It can be conceptualized in terms of actual or perceived support, and includes diverse functionalities, such as instrumental or emotional support. Social support can be especially important for marginalized populations or individuals facing vulnerabilities. Interventions seeking to strengthen ties within a social network or expand someone’s own social network focus on impacting downstream psychosocial mechanisms related to health, such as social support, social influence, and access to social and material resources.

We sought to systematically review the published literature to ascertain how social tie formation and/or strengthening has been used for HIV prevention interventions and whether such interventions have been effective in reducing quantitatively measured HIV-related outcomes in low- and middle-income countries. Secondary aims were to develop a typology of how social tie interventions work to impact HIV prevention and describe how interventions have measured changes in social ties.

We undertook this review with full knowledge that literature on social ties and health is fraught with complex theories, ambiguous concepts, and often conflicting definitions and applications; hence this review also sought to better understand how these concepts are being applied and operationalized within interventions.

## Methods

This review was conducted as part of a larger systematic review initiative, The Evidence Project, which seeks to understand effectiveness of behavioral and structural interventions related to HIV prevention in low- and middle-income countries. This review employed standardized protocols and procedures used in previous reviews [22] and followed standard systematic review guidelines set forth in the Preferred Reporting Items for Systematic Reviews and Meta-Analyses (PRISMA) statement [23].

### Definition and Inclusion criteria

We included studies conducted in a low- or middle-income country as defined by the World Bank; published in a peer-reviewed journal from January 1, 1990 through the search date of August 16, 2022; involved interventions explicitly focused on building/strengthening social relationships; contained relevant health-focused outcomes pertaining to HIV and/or sexual behavior; and presented pre/post or multi-arm quantitative results. A social “tie” was defined as a social connection that elicits mutual feelings of trust, reciprocity, and recognition of shared identity or increases access to shared information or resources. For inclusivity, we intentionally crafted a definition devoid of terms specific to any particular social theory. If a study mentioned using a specific social theory, such as social capital, the intervention was only included if authors explicitly described it as attempting to form or strengthen social ties, regardless of whether the authors used that term. We defined social tie building interventions as any intervention which sought to: (1) create a group with the intention of strengthening ties between group members; or (2) strengthen existing ties within a group; or (3) strengthen ties between groups/individuals or strengthen ties among non-peers.

We excluded interventions focused on using *existing* social ties or networks to disseminate information or change social norms, such as through peer education, as these interventions do not attempt to alter social relationships themselves. We also excluded studies focused on building or altering familial ties, such as between parents and children or between spouses. Although these relationships are important to health throughout the life course, we focused on social ties external to family relationships as these may be most amenable to change in programmatic contexts. Given our focus on HIV prevention, we excluded social tie building interventions exclusively among people living with HIV as these interventions typically involve building social ties for purposes of strengthening social support, promoting medication adherence, and improving mental health and coping. While it is possible that these support-building interventions among people living with HIV could have reduced the transmission of HIV through promoting viral suppression, this topic has been covered in several recent reviews [24, 25]. Our focus was on primary prevention of HIV among HIV-negative individuals. We also excluded studies that built or strengthened social ties but did so using someone in a paid position, such as a peer navigator.

### Search Strategy

We searched five electronic databases, including PubMed, CINAHL, EMBASE, Sociological Abstracts, and PsycINFO using the following search terms:

(“social capital” OR “social network” OR “social participation” OR “social cohesion” OR “social inclusion” OR “social involvement” OR “social action” OR connectedness OR “group cohesion” OR “group participation” OR “group inclusion” OR “group involvement” OR “group membership” OR “community building” OR “community participation” OR “community cohesion” OR “community inclusion” OR “community involvement” OR “collective action” OR “community organizing” OR “community mobilization” OR “community mobilisation” OR “empowerment” OR “community group” OR “capacity building” OR “political activism” OR “political advocacy” OR “unionization” or “unions”) AND (HIV OR AIDS OR HIV/AIDS).

We excluded titles and abstracts containing reference to high-income settings given our focus on low- and middle-income countries. The titles and abstracts from initial search results, with duplicates removed, were screened first by trained research assistants to remove irrelevant citations, and remaining citations were screened independently by two study team members to determine eligibility, with differences resolved through consensus. Full text articles were obtained and screened for citations in which eligibility could not be determined through title and abstract review alone.

In addition to the database search, we also examined the table of contents of HIV-specific journals over the same search period, including *AIDS*, *AIDS and Behavior*, *AIDS Care*, and *AIDS Education and Prevention*, and we iteratively searched reference lists of all included articles to identify additional studies.

### Data Extraction and Analysis

Data were extracted using standardized forms, which included the following components: study location, eligibility criteria, population, sample size, sampling, study design, intervention characteristics, outcomes (including effect sizes and statistics), and additional information, such as costs, community acceptance, and limitations. Data were extracted in duplicate by two trained staff. Differences were resolved through consensus among extractors, with additional study team input when needed. Data were entered into REDcap (Research Electronic Data Capture) [26, 27] and exported into Excel.

We evaluated the methodological rigor of studies using a validated risk of bias tool developed to assess the risk of bias across randomized and non-randomized study designs [28], which has been used previously for similar reviews focused on social and behavioral interventions related to HIV prevention [17, 29–36]. It comprises the following criteria: (1) following a cohort of individuals, (2) having a control and intervention/experimental group, (3) comparing baseline sociodemographic characteristics between groups, (4) comparing baseline outcomes between groups, (5) reporting pre- and post-intervention data, (6) random selection of participants for assessment, (7) random assignment of participants to intervention, and (8) follow-up rates [28].

**Fig. 1:**
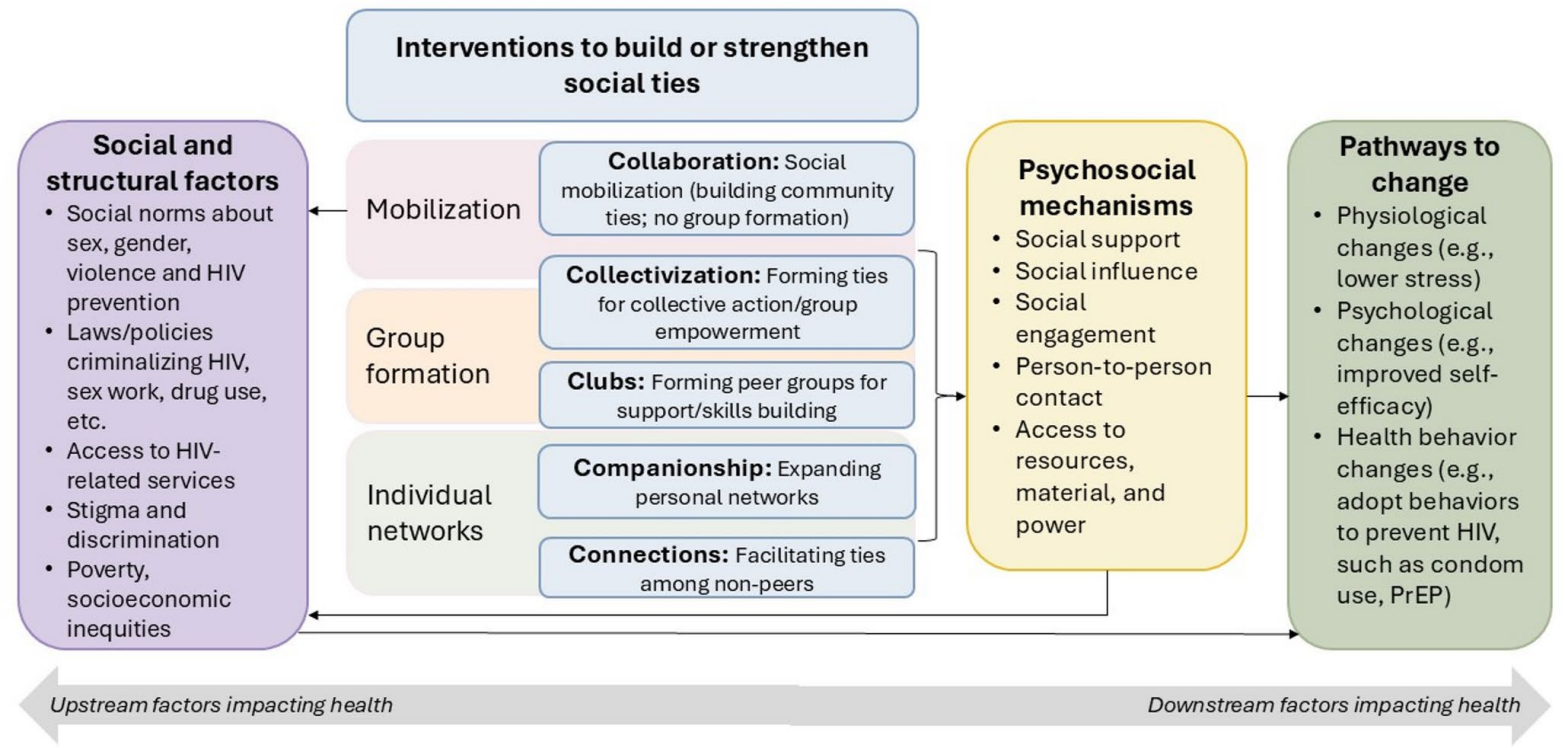
Conceptual model of types of social tie interventions and how they impact health

Results were synthesized qualitatively. Quantitative synthesis was infeasible given differences in study designs and outcomes. We also developed a typology to categorize interventions by type of social tie intervention. Initially, studies were categorized based on the theoretical underpinnings provided within the intervention description. However, given both a lack of consistency in how theories were applied and an often lack of explicit theory provided, we instead categorized interventions by how they used social ties to achieve their stated goal. To accomplish this, we identified existing relevant frameworks in the literature, including Berkman and Glass’s conceptual model of how social networks impact health [37], and a typology developed by Gram et al. to categorize community-based interventions [38]. From these frameworks we developed a conceptual model (Fig. 1), inclusive of functional categories of social tie strengthening interventions. The Berkman and Glass framework outlines pathways through which social networks impact health, including through upstream and downstream pathways. We retained these pathways in our conceptual model and added specific types of social tie strengthening interventions adapted from the Gram et al. typology. In the Gram et al. typology, community-based interventions were classified into three categories: “classroom,” “collectives,” and “clubs.” For our current typology, we dropped the “classroom” categorization given the lack of focus on social tie-building (i.e., these were defined as didactic interventions with group-based platforms), built upon definitions of “collectives” and “clubs,” and added three additional categories: “collaboration,” “companionship,” and “connection.” We developed definitions for each type of intervention (Table 1) and two study staff classified included studies based on these categories, with differences resolved through consensus. Notably, some interventions involved components related to social ties that cut across categories (i.e., the interventions involved more than one type of social tie building activity), thus we have noted cross-cutting studies as a separate category. Definitions and examples of each category are presented in Table 1.

**Table 1:** Social tie building/strengthening intervention typology

Category	Definition	Pathways for change
**Collaboration**: Community or social mobilization	Interventions that seek to strengthen social ties within communities as part of community-wide mobilization campaigns but do not involve forming or strengthening specific groups within a community.	Primarily aimed at altering upstream social and structural factors (e.g., norms, policies or laws, healthcare access, stigma, socioeconomic inequalities).
**Collectivization**: Forming ties for collective action and/or group empowerment	This type of intervention is often similar in purpose to social mobilization, but in these instances the focus is on building ties within a specific group. Most often these interventions focus on building ties within a group of individuals from marginalized communities, such as sex workers, specifically to help facilitate collective action and empowerment at the group and/or individual-level.	Aimed at altering upstream social and structural factors, as well as downstream psychosocial processes (e.g., social support, social engagement, access to resources).
**Clubs**: Forming peer groups for support and skills building	This type of intervention seeks to create or strengthen peer groups, specifically to facilitate peer-based support. Often these interventions involve training on some sort of skill, such as income generating skills, communication skills, etc. The difference between this category and collectivization is that there is little to no mention of collective action or empowerment within these “clubs.” In other words, the emphasis is mostly on downstream health-related factors (i.e., psychosocial support), not upstream factors (i.e., structural change).	Seeks to alter downstream psychosocial processes, such as enhancing social support. May seek to provide skills, such as income generating skills, to address specific structural determinants.
**Companionship**: Expanding personal networks	In this category, the goal is not to build or strengthen a group of individuals, but instead focuses on building the personal networks of specific individuals (without formation of a group). The purpose of the network expansion could be to find peers who share a characteristic that might make someone marginalized (such as helping gay men find other gay men to build support networks), or to expand someone’s network to individuals who do not share a specific characteristic (e.g., connecting people who use drugs to people who do not use drugs).	Seeks to alter downstream psychosocial processes, such as enhancing social support, social engagement, and access to resources.
**Connections**: Facilitating ties among non-peers	This category is similar to the concept of “bridging” social capital in that these interventions attempt to bring together individuals from different levels of power, experiences, and duties/roles to forge common understandings, innovation, and access to ideas/resources that otherwise would have been inaccessible without creating these ties and relationships	Seeks to alter downstream psychosocial processes, especially access to resources and spheres of social influence outside of one’s typical circles.

## Results

### Description of Included Studies

**Fig. 2:**
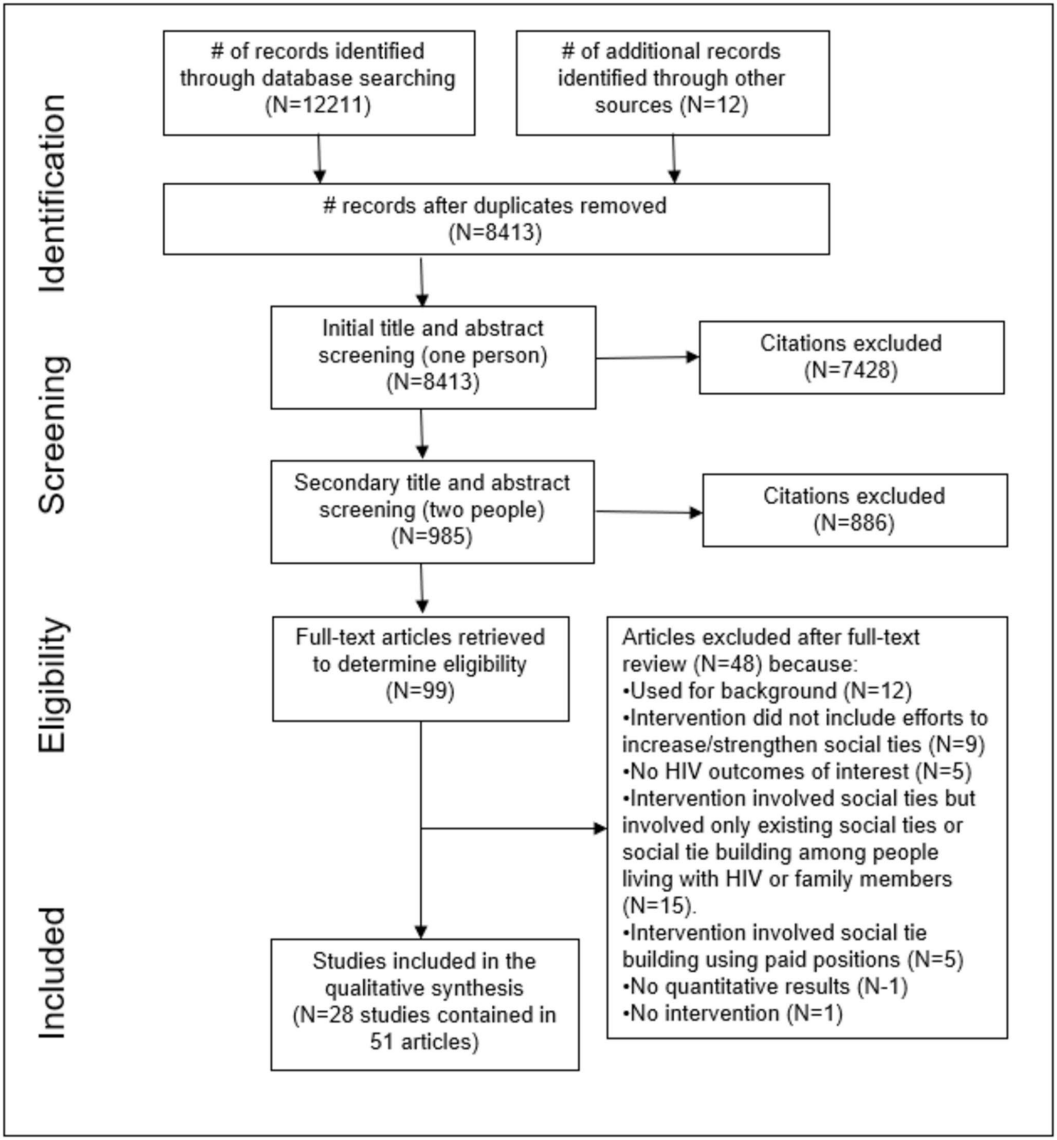
PRISMA flowchart depicting disposition of citations

The search yielded 8414 unique citations, and after initial screening, 100 citations were retained to determine eligibility (Fig. 2). Of these, 51 articles covering 28 studies were included. Studies comprised diverse populations, including community members of geographically defined spaces (e.g., villages, districts) (*n* = 4) [39–46], sex workers (*n* = 16) [10, 47–78], men who have sex with men (MSM) (*n* = 3) [78–80], people who use drugs (*n* = 2) [81, 82], youth (*n* = 5) [83–87], and married women living in rural areas (*n* = 1) [88]. Studies took place across sub-Saharan Africa (*n* = 11) (including South Africa (*n* = 2) [40, 42–44], Tanzania (*n* = 1) [76], Uganda (*n* = 2) [39, 41, 45], Kenya (*n* = 1) [55], Zambia (*n* = 1) [87], Zimbabwe (*n* = 2) [49, 75], Ghana (*n* = 1) [83]), and one multi-country study [85, 86]); Asia (*n* = 12) (including India (*n* = 6) [10, 47, 50, 51, 56, 58–74, 78], China (*n* = 2) [79, 81], Thailand (*n* = 3) [82, 84, 88], and The Philippines (*n* = 1) [57]), and the Americas/Caribbean (*n* = 5) (including Brazil (*n* = 3) [48, 53, 54, 77], The Dominican Republic (*n* = 1) [52], Mexico (*n* = 1) [80]). Table 2 contains characteristics of included studies.

**Table 2:** Description of included studies

Intervention	Setting	Population	Intervention Description	Study Design	Key outcomes and results
**Collaboration**: Social mobilization interventions					
SASA! [39, 41]	Kampala, Uganda	Community members	The intervention aimed to change community attitudes, norms and behaviors related to gender inequality, violence, and increased HIV vulnerability for women. It advanced new concepts of power and encouraged scrutiny of power imbalance through activities such as advocacy, activism, media, training, and others. Community activists and leaders were supported to conduct local activism activities, including door-to-door visits, interactive community dramas, film shows, poster discussions, public events and ‘quick chats.’	Group randomized trial	*Positive change*: lower sexual intimate partner violence (IPV) for women; men had increased condom use, fewer sexual concurrency, increased HIV testing
SHARE (Safe Homes and Respect for Everyone) [45]	Rakai, Uganda	Community members	The violence reduction intervention aimed to reduce physical and sexual intimate partner violence (IPV) and HIV incidence. The intervention consisted of five phases based on the stages of change theory: community assessment, raising awareness, building networks, integrating action, and consolidating efforts.	Evaluated within existing cluster randomized cohort	*Positive change*: reduced HIV incidence; fewer reports of physical IPV *No change* in male perpetration of IPV
Adapted “One Man Can” campaign [42, 46]	Mpumalanga Province, South Africa	Community members	The community mobilization intervention aimed to increase awareness of gender inequities and HIV and encourage community members, especially boys and men, to take action to address negative gender norms and associated HIV risk. Local community mobilizers and volunteer cadres delivered workshops, created opportunities for community dialogue through community activities (door-to-door home visits, street soccer, etc.), and facilitated leadership engagement and support on intervention content and activities.	Group-randomized trial	*Positive change*: more equitable gender norms (among men), fewer sex partners (among women) *No change*: HIV testing, condom use, gender norms for women, perpetration of IPV
**Collectivization**: Forming ties for collective action and/or group empowerment					
Sonagachi Project [10, 50]	West Bengal, Calcutta, Sonagachi District, India	Sex workers	The aim was to empower sex workers to promote sexual behavior change and improve health. The approach focused on respect, resilience, and recognition. Sonagachi sex workers were exposed to trained peer educators (also sex workers). Peer educators engaged in community-based information, education, and communication activities related to HIV/STDs, including condom promotion and clinic referral.	Serial cross sectional; Cross-sectional	*Positive change*: increased treatment-seeking behaviors, HIV testing *No change*: STI prevalence
Sonagachi Project Replication [47]	Cooch Behar, Dinhata, India	Sex workers	The aim was to build skills and confidence in providing education and to foster empowerment and advocacy for local sex workers to effect changes in structural barriers to condoms. The intervention included community organizing and advocacy, peer education, condom social marketing, and establishment of a health clinic.	Group randomized trial	*Positive change*: increased condom use
Sonagachi Project Replication [56]	West Bengal, Calcutta, Sonagachi District, India	Sex workers	The aim was to reduce vulnerability to HIV/STD infection through empowerment strategies (community organizing and mobilization, rights-based framing, advocacy, micro-finance). STD clinics were established in both communities as standard of care, including in-clinic peer education and condom social marketing. Empowerment intervention strategies (community organizing, advocacy, rights-based framing, micro-finance) were implemented	Group randomized trial	*Positive change*: workplace skills (e.g., condom negotiation), knowledge of risk and protective factors, social support via organizing/solidarity *No change*: Stigma, voting to build social capital
Avahan Iniative [58–71]	India Andhra Pradesh, Karnataka, Maharashtra, Tamil Nadu	Female sex workers	The main objective was to deliver a rapidly scaled prevention program to high-risk groups. The program’s strategies were designed to achieve high coverage in selected geographic areas through delivering a combination package of prevention services addressing proximal and distal determinants of HIV risks. Key elements included: peer-led outreach and education; free condoms; STI treatment services; referrals for HIV and TB testing, and HIV care; empowerment and community mobilization to address local structural barriers; and mass communication programs. Other services, including condom and STI treatment social marketing and behavior change communication, were targeted specifically to clients of FSWs.	Cross sectional or serial cross-sectional design	*Positive change*: increased condom use; analyses highlighted that collective action, collective agency, and individual self-efficacy play mediating role in health-related outcomes
Avahan Initiative– Project Parivartan [72–74]	Rajahmundry area of the East Godavari district in Andhra Pradesh, India	Female sex workers	The intervention organized FSWs to build collective power and channel this power to address structural determinants of HIV risk, including stigma, policing policies and practices, condom availability, and access to STI testing and treatment and to loans to address economic vulnerability. It encouraged FSWs to form FSW-led community-based organizations. It also used ‘social change agents’, local FSWs who serve as peer health educators and community organizers. FSWs mobilized by the intervention held public rallies and media events, met with public officials and community groups to promote awareness of FSW issues, assisted with intervention-run STI clinics and responded to FSW complaints of police mistreatment.	Cross sectional or serial cross-sectional design	*Positive change*: increased condom use
Empowerment for MSM [80]	Mexico	Gay and bisexual men in Mexico	The aim was to promote HIV preventative behaviors through various empowerment strategies. Individuals attended regular meetings on HIV prevention and worked together to develop plans to enhance their ability to be health educators and promote community-based HIV education. Planning activities included regular group discussion sessions, and planning activities for its community AIDS prevention program. Weekly sessions included discussion topics on health education, outreach, and group initiatives.	Prospective cohort	*Positive change*: increased condom use, HIV knowledge *No change*: number of recent sexual partners
100% Condom Campaign [52]	Santo Domingo, Puerto Plata, Dominican Republic	Establishment-based female sex workers	The aim of the environmental-structural intervention was to reduce risk of HIV and STIs among female sex workers. The intervention included workshops on solidarity & collective commitment, environmental cues (media campaigns in establishments, clinical services, and monitoring/encouraging adherence). The second intervention group also received policy and regulation (sex work venues required to procure/provide condoms).	Serial cross-sectional	*Positive change*: increased condom use and rejection of unsafe sex, decreased number of sexual partners and STIs
Belgaum Integrated Rural Development Society (BIRDS) [51]	Karnataka, India	Female sex workers in Karnataka	The intervention aimed to empower FSWs and increase their capacity to negotiate safer sexual relationships. Peer educator activities include awareness campaigns, dissemination of culturally appropriate educational materials, expansion of use and distribution of condoms and ongoing recruitment of collective members. The collectivization process also introduced literacy training, legal assistance, health care and other services.	Cross-sectional study	*Positive change*: higher collectivization linked to increased condom use and HIV knowledge
Projeto Princesinha [48]	Manacapuru, Brazil	Female sex workers	The objective was to stop STD/HIV transmission and expand access to diagnosis and treatment of incident cases in the sphere of the Sistema Único de Saúde, valuing principles of social control, self-sustainability and reproducibility. Female sex workers were chosen and trained as peer educators to promote and sell low-cost condoms, refer sex workers to care for STDs, educate sex workers, clients, and school children about HIV/STDs, and track condom retail. Community activities were held to legitimize sex workers in the community.	Serial cross-sectional	*Positive change*: increased condom use and condom sales, reduced number of sexual partners, increased HIV testing
Fio da Alma [53]	Rio de Janiero, Brazil	Female sex workers	The intervention aimed to integrate community development activities into an ongoing HIV/STI peer education program. Peer educators were trained and encouraged to meet regularly with sex workers to discuss issues of common concern regarding health/well-being, and to encourage participation in the sex worker organization, Fio da Alma. A Drop-in-Center was opened to create a safe space for sex workers to come together, hold workshops/activities and serve as project offices. Sex workers were asked to jointly identify priority action areas and then solicited small funds and technical assistance from NGO project staff to implement activities. Condoms, health education and health/social services were available.	Serial cross-sectional	*Positive change*: social cohesion and mutual aid associated with increased condom use *No change*: No change in overall HIV/STI protective behaviors (limited intervention participation)
Frontiers Prevention Project (FPP) [78]	Andhra Pradesh, India	Men who have sex with men, Female sex workers	The intervention aimed to improve advocacy within key populations, change policies, and increase community awareness. A comprehensive package of prevention interventions was provided, including: STI services, behavior change communication, condom programs, community mobilization, and enabling and structural interventions. There was emphasis on social capital building, network and support formation, empowerment, violence reduction, referrals for HIV testing and care.	Serial cross-sectional	*Positive change*: increased condom use and decreased STI prevalence
Encontros [54, 77]	Corumba, Brazil	Sex workers	The project aimed to engage sex workers on an individual level through participation in STI/HIV counseling and testing, on an interpersonal level through peer-education, and on a community level through outreach and social activities. Designed community-based activities to extend and strengthen collegial relationships through providing sex workers opportunities to engage in dialogue around sex work, discrimination, human rights, and prevention.	Prospective cohort; cross-sectional	*Positive change*: intervention exposure associated with increased condom use, social cohesion, and network participation *No change*: Access to resources, non-significant decline in STI incidence
Kapihan [57]	Metro Manila, Philippines	Sex workers	The 4-hour intervention aimed to reduce sexual risk and increase HIV testing among sex workers by integrating human rights with HIV skill-building and community mobilization. It provided HIV and STI education and condom demonstrations and human rights education components focused on raising participant knowledge and understanding of laws protecting rights of individuals against abuse. To build community/mobilization momentum, facilitators conducted an aspiration/goal-building exercise for individuals and the larger group.	Before-after study	*Positive change*: knowledge of HIV/STIs, intentions to test for HIV
Shikamana [76]	Iringa, Tanzania	Female sex workers	The community empowerment combination prevention intervention included a community-led drop-in-center to build social cohesion and host mobilization activities; venue-based peer education, condom distribution and HIV counseling/testing; peer service navigation and social support to promote HIV treatment access and adherence; HIV care provider and police sensitivity trainings; and text messages to promote solidarity and intervention engagement, as well as reminder messages to promote care engagement/treatment adherence.	Prospective community-randomized trial	*Positive change*: reduced HIV incidence, increased condom use and HIV behavioral care outcomes *No change*: nonsignificant increases in viral suppression
**Clubs**: Forming peer groups for support and skill building					
HIV prevention for married women [88]	Chiang Mai, Thailand	Married women	The intervention aimed to help married women improve self-efficacy, self-esteem, and their behavioral skills for negotiation of safe sex practices and condom use. The intervention also sought to mobilize communities through grass roots initiatives and local leadership to establish informal organizations and networks for HIV prevention interventions. The intervention also involved sessions held for married women on increasing HIV knowledge, condom use and practice skills, and building safe sex and negotiation skills.	Group randomized trial	*Positive change*: increased condom use and condom negotiation skills
Microenterprise services among sex workers [55]	Nairobi, Kenya	Female sex workers	The intervention aimed to add micro-enterprise services to a peer-mediated HIV/AIDS intervention that entailed providing credit for small business activities, business skills training and mentorship, and promotion of a savings culture among sex workers.	Before-after study	*Positive change*: reduced number of weekly sexual partners, increased condom use
Peer network among methamphetamine users [82]	Chiang Mai, Thailand	Young adults who use methamphe-tamine	The intervention aimed to reduce methamphetamine and HIV risk behaviors. Participants received seven, 2-hour small group sessions meeting twice weekly, which taught participants to think critically about and reduce drug use and sexual risk behaviors. Participants developed and practiced communication skills to create risk reduction messages for their networks. Sessions comprised role play, interactive teaching modules, instructive games and problem-solving activities, and a community service project chosen by the group.	Randomized trial- individual	*Positive change*: increased condom use, decreased methamphetamine use
Adolescent Girls Empowerment Program [87]	Zambia– 4 provinces (unspecified)	Most vulnerable never-married adolescent girls.	The intervention aimed to address challenges facing girls like early childbearing, unintended pregnancy, gender-based violence, HIV etc. by building social, health, and economic assets in the short term and improving sexual behavior, early marriage, pregnancy, and education longer term. One component was a weekly girl group meeting facilitated by a trained young, female mentor, where curricula-guided sessions include sexual and reproductive health, HIV, life skills and financial education. Another component provided health vouchers for girls to go to health service providers for free general and sexual/reproductive wellness and care. The third component involved opening an adolescent-friendly savings account.	Multi-arm randomized cluster design	*Positive change*: increased SRH knowledge, financial literacy, savings behavior, self-efficacy; less transactional sex *No change*: education outcomes, fertility outcomes, gender equity norms, HIV knowledge and acceptability of IPV
**Companionship**: Expanding personal networks					
Relational model for HIV prevention among drug users [81]	Guangxi, China	Women who inject drugs	The intervention used an indigenous culture-based model for use by NGOs to prevent HIV among people who inject drugs. Participants received HIV education from a prevention worker who focused on building trust through giving a small gift and dialogue. The prevention worker also spoke with family members about how to support the participant’s health and help her build a support group of non-drug using friends. Police agreed not to disrupt the intervention, and reduced rate health care was available to participants and their families. HIV education was provided to the community at trainings/events.	Before/after study design	*Positive change*: increased HIV knowledge and, condom use, decreased needle sharing
Gay bar-based Participatory Entertainment-Education (PEE) [79]	Chengdu, China	Gay men, Men who have sex with men, and money boy commercial sex workers	The intervention aimed to use socially and culturally appropriate participatory communication to promote safer sex behaviour with gay men and MSM. The intervention included many participatory activities conducted to develop safer sex cultural practices among gay men/MSM within various gay venues, events and networks (e.g., a gay-bar based melodrama series on condom use in the gay community; media materials; outdoor edutainment and group discussions).	Individual non-randomized trial	*Positive change*: increased condom use
**Connections**: Facilitating ties among non-peers					
Youth-friendly sexual and reproductive health intervention [83]	Kassena-Nankana, Ghana	Adolescents	The social learning intervention aimed to improve adolescent access to and usage of sexual and reproductive health services through: (1) community mobilization; (2) health worker training in youth-friendly approaches, including activities promoting adolescents and provider interaction; (3) school-based sexual health education; (4) out-of-school peer outreach.	Group randomized trial	*Positive change*: increase in use of STI and antenatal services
C2P (Connect 2 Protect) adaptation [84]	Chiang Mai, Thailand	Youth (adolescents and young adults 14–24)	The intervention aimed to prevent methamphetamine use among youth and reduce HIV risk behaviors through community structural changes. Through a community mobilization approach, coalitions composed of local leaders from various sectors were formed to develop and implement structural changes at the community level.	Group randomized trial	*No change*: no difference in lifetime methamphetamine use (nonsignificant decrease)
**Cross-cutting interventions** (combining social mobilization with peer group formation)					
Intervention with Microfinance for AIDS and Gender Equity (IMAGE) [40, 43, 44]	Limpopo Province, South Africa	Women and communities	The IMAGE intervention combined a poverty-focused microfinance initiative targeting the poorest women in communities with a participatory curriculum of gender and HIV education. In the group-based microfinance component, groups of women received loans to establish small businesses. Further credit was offered when all women per group repaid their loans. The second component comprised a gender and HIV training curriculum integrated into established biweekly meetings of 40 women for approximately 1 year.	Group randomized trial	*Positive change*: decrease in IPV experienced by women *No change*: HIV incidence and condom use with casual partner
DREAMS [85, 86]	Nairobi and Gem, Kenya uMkhanyakudaSouth Africa	Adolescent girls and young women aged 10–22 years	The aim was to deliver combinations of biomedical, behavioral, and structural interventions to reduce HIV incidence among adolescent girls and young women through a public-private sector partnership.	Prospective cohort	*Positive chance*: increased social support, self-efficacy
SAPPH-IRe [49]	Zimbabwe (national)	Female sex workers	The community mobilization intervention activities facilitated by clinical staff aimed to raise awareness of the benefits of ART and PrEP, strengthen support networks to encourage health-promoting behavior, and build leadership skills. A “sister”, nominated by ART and PrEP users, was responsible for supporting adherence and attending program training sessions with participants. Clinical services were also improved to support on-site initiation of ART and PrEP.	Group-randomized trial	*Positive change*: increased HIV testing and HIV diagnosis *No change*: Proportion of sex workers with viral load < 1000 copies per ml
Sisters with a Voice [75]	Mutare, Victoria Falls, and Hwange, Zimbabwe	Female sex workers	The intervention intensified community mobilization providing peer-delivered activities to foster an enabling environment for sex workers to adopt protective behaviors and increase use of health care services. Peer educators brought FSWs together in participatory workshops to build solidarity and reduce competition. Public sector health workers were trained to be more ‘sex worker friendly’ through a workshop and shadowing program with nurses at the Sisters’ clinics and having monthly meetings with staff and peer educators.	Time series	*Positive change*: higher proportion of HIV-positive sex workers who knew their HIV status and reported being on ART; increased HIV testing among HIV-negative sex workers *No change*: condom use with regular partners

### Study Categorizations, Theory, and Social Tie Measures

We sorted studies into five functional intervention categories that sought to build or strengthen ties (Fig. 1): (1) **Collaboration**: strengthening ties through social mobilization (*n* = 3 ); (2) **Collectivization**: forming ties for collective action/group empowerment (*n* = 13); (3) **Clubs**: forming peer groups for support/skills building (*n* = 4); (4) **Companionship**: expanding personal networks with peers (*n* = 2); (5) **Connections**: strengthening ties between heterogenous groups or individuals (*n* = 2). Additionally, 4 studies included multiple intervention components that addressed two or more different types of social tie strengthening; thus, these have been included as cross-cutting interventions. Below we discuss studies identified within each category.

Across categories, studies described theoretical underpinnings mostly aligned with community mobilization [39–41, 43–45, 49, 52, 57, 84, 88], empowerment [10, 47, 50, 51, 55, 56, 78, 80], or both [58–74, 76]. Examples of other theories mentioned included Social Learning Theory [82, 83, 88]), ecological model [54, 77], Community Development [53], participatory approaches [79], and social capital [40, 43, 44].

Of 28 included studies, 12 (43%) attempted to measure constructs relevant to social ties (Table 3). These measures differed by study and related to theoretical components of how social ties were being altered and how this would lead to change. Some common constructs were measures of social cohesion [42, 53, 54], participation in collective action or mobilization activities [42, 43, 56, 57], and both individual- and group-level empowerment [59, 63, 64, 71, 72].

**Table 3:** Underlying theories and measurement of social ties constructs

Study (Citation)	Theory underlying intervention	Construct(s) related to social ties	Measurement of social tie construct
***Collaboration***: *Social mobilization interventions*			
SASA! [39, 41]	Community mobilization, ecological model of violence	Not reported	Not reported
SHARE [45]	Community mobilization; Stages of Change	Not reported	Not reported
One Man Campaign [42]	Community mobilization	Community Mobilization Measure comprising 6 constructs: Social cohesion, critical consciousness, shared concerns, leadership, organizational networks, collective action	o Social cohesion: Community connectedness and working trust o Critical consciousness: Consciousness and critical thinking, including whether the community is undergoing processes of critical reflection and dialogue about shared circumstances and finding solutions that address injustices o Shared concerns: Whether community members define HIV as an important and mutable issue, and whether they are aware of the impact of HIV in their village and believe they can work together to prevent HIV o Leadership: Leadership capacity, diversity, responsiveness, accessibility, and support of democratic or collective decision-making o Organizational networks: The existence of community-based organizations, groups, and networks that can serve as a resource for mobilizing o Collective action: Presence, breadth, and quantity of collective activities in villages aimed at social change
***Collectivization***: *Forming ties for collective action and/or group empowerment*			
Sonagachi Project [10, 50]	Empowerment	Not reported	Not reported
Sonagachi replication [47, 56]	Empowerment	Social support via organizing and solidarity; political participation	o Social support via organizing & solidarity (Visited with sex workers outside work, participated in social functions, helped other sex workers) o Political participation for social capital (Voting), described as proxy for building social capital
Avahan [58–71]	Community mobilization; individual and collective empowerment	Collective empowerment	o Power within: Relating to self-esteem and confidence o Power with: Relating to collective identity and solidarity o Power over: Relating to access to social entitlements
Avahan– Project Parivartan [72–74, 74]	Empowerment; community mobilization	Collective identity, efficacy, and agency	o Collective identity- identification with other FSW o Collective efficacy- belief that FSW can work together for change o Collective agency- action with other FSW on behalf of FSW o Blankenship et al. assessed composite empowerment indicators through assessing different forms of power, including power within (ability to build capabilities such as self-esteem, confidence, and agency), power over resources (ability to access social entitlements and micro-credit), and power with (through formation of collective identities)
Empowerment for MSM [80]	Empowerment	Not reported	Not reported
100% Condom Campaign [52]	Community mobilization	Not reported	Not reported
BIRDS [51]	Empowerment	Collectivization index	o Classified into high, medium and low levels of collectivization based on: (1) being a member of local collective and (2) contact with peer educator in past year
Projeto Princesinha [48]	Community mobilization and reference to Paulo Freire	Not reported	Not reported
Fio da Amo [53]	Community development	Social cohesion, social participation, access to social and material resources	o Perceived social cohesion and mutual aid among sex workers from same area (8 items): level of group connectedness and caring and potential for instrumental, emotional and material support. o Participation in secondary associations: (5 items): neighborhood organizations, church groups, community centers, health groups and social clubs. o Access to and management of personal material resources (12 items): bank account, savings plan, social security contributions, health insurance and purchasing power
Frontiers Prevention Project [78]	Empowerment	Not reported	Not reported
Encontros [54, 77]	Empowerment and ecological model	Social cohesion, participation in network, access to social and material resources	o Social cohesion: perception of mutual aid, trust, connectedness, support among sex workers o Participation in networks: access to and participation in social and community networks o Access to and management of material and social resources, including goods and services
Kapihan [57]	Community mobilization	Community mobilization	o Have you participated in community mobilization activities around your health and human rights as sex workers or entertainers in the past 6 months? o Would you participate in an organization that addresses health and human rights of sex workers in the next 3 months?
Shikamana [76]	Empowerment	Not reported	Not reported
***Clubs***: *Forming peer groups for support and skills building*			
HIV prevention for married women [88]	Community development, community mobilization, Miller’s Theory and Social Learning Theory	Not reported	Not reported
Microenterprise services among sex workers [55]	Empowerment	Not reported	Not reported
Peer network among methamphetamine users [82]	Social learning theory, social identity theory, Diffusion of Innovations	Not reported	Not reported
AGEP Program [87]	Empowerment (social, health, and economic assets)	Availability of safe spaces	o Had a safe space in the community to meet with friends- whether the girl had a safe space in the community to meet with friends
***Companionship***: *Expanding personal networks*			
Relational model for HIV prevention among drug users [81]	Community mobilization and Confucianism	Not reported	Not reported
Edutainment for MSM [79]	Empowerment, ecological model, participatory approach, Health Belief Model, Social Cognitive Theory, Theory of Reasoned Action	Network expansion	o Number of friends who are gay men homosexual men (MSM) o Amount of free time spent with gay men or MSM
***Connections***: *Facilitating ties among non-peers*			
Youth-friendly sexual and reproductive health intervention [89]	Community mobilization, Social Learning Theory	Not reported	Not reported
C2P (Connect 2 Protect) adaptation [84]	Community mobilization	Not reported	Not reported
***Cross-cutting interventions***: *Combining social mobilization with peer group formation*			
IMAGE [40, 43, 44]	Social capital, microfinance	Social capital	o More participation in social groups o Taken part in collective action o Greater perception of community support in a time of crisis o Belief that the community would work together toward common goals o More positive attitude to communal ownership
DREAMS [85, 86]	Empowerment, community mobilization	Social support Social asset and social protection activities uptake	o Social support: A binary, composite variable summarizing social support, including social connectedness, was created using four questions on female networks and access to safe social spaces to meet o Social asset building activities included: Building social skills and networks, connecting with AYW peers and adults for informational, emotional and material support and their update was analyzed o Social protection activities included: Cash transfers, educational subsidies, combination socio-economic approaches. Uptake of all these activities by participants was analyzed.
SAPPH-Ire [49]	Community mobilization	Not reported	Not reported
Sisters with a Voice [75]	Community mobilization	Not reported	Not reported

### Risk of Bias

Table 4 presents the risk of bias across studies. Studies encompassed a wide range of designs and rigor. Among experimental designs, group randomization trials were most common. Among quasi-experimental methods, designs included cohort studies, serial cross-sectional studies, and cross-sectional studies, typically measuring a dose-response relationship (i.e., comparing varying degrees of intervention exposure in relation to outcomes of interest). Notably, all “collaboration” studies were randomized group trials whereas the vast majority of “collectivization studies” employed quasi-experimental means, although there were notable exceptions. Studies examining interventions in remaining categories applied a mix of experimental and quasi-experimental designs.

**Table 4:** Risk of bias assessment table

Study	Author Year	Cohort	Control or comparison group	Pre/post intervention data	Random assignment of participants or groups to the intervention	Random selection of participants for assessment	Follow-up rate of 80% or more	Comparison groups equivalent at baseline	
								In socio-demographics	In outcome measures
***Collaboration***: *Social mobilization interventions*									
SASA!	Abramsky 2014	No	Yes	Yes	Yes	Yes	N/A	Yes	NR
	Kyegombe 2014	No	Yes	Yes	Yes	Yes	N/A	Yes	NR
SHARE	Wagman 2015	Yes	Yes	Yes	Yes	Yes	No	Yes	No
One Man Campaign	Lippman 2017	No	Yes	Yes	Yes	Yes	NA	Yes	NR
	Pettifor 2018	No	Yes	Yes	Yes	Yes	NA	Yes	No
***Collectivization***: *Forming ties for collective action and/or group empowerment*									
Sonagachi	Jana 1998	No	No	Yes	N/A	No	N/A	N/A	N/A
	Gangopadhyay 2005	No	Yes	No	No	Yes	N/A	No	No
Sonagachi replication	Basu 2004	Yes	Yes	Yes	Yes	Yes	Yes	No	No
	Swendeman 2009	Yes	Yes	Yes	Yes	Yes	Yes	NR	NR
Avahan	Adhikary 2012	No	No	Yes	N/A	Yes	N/A	N/A	N/A
	Blanchard 2013	No	No	Yes	N/A	Yes	N/A	N/A	N/A
	Boily 2013	No	No	Yes	N/A	Yes	N/A	N/A	N/A
	Deering 2011	No	No	No	N/A	Yes	N/A	N/A	N/A
	Fehrenbacher 2016	No	No	No	No	No	N/A	N/A	N/A
	Guha 2012	No	No	No	N/A	Yes	N/A	N/A	N/A
	Kuhlmann 2014	No	No	No	No	No	N/A	N/A	N/A
	Mainkar 2011	No	No	Yes	N/A	No	N/A	N/A	N/A
	Rachakulla 2011	No	No	Yes	N/A	Yes	N/A	N/A	N/A
	Ramakrishnan 2010	No	No	No	N/A	Yes	N/A	N/A	N/A
	Ramesh 2010	No	No	Yes	No	Yes	N/A	N/A	N/A
	Reza-Paul 2008	No	No	Yes	No	Yes	N/A	No	N/A
	Thilakavathi 2011	No	No	Yes	N/A	Yes	N/A	N/A	N/A
	Yadav 2013	No	No	No	No	No	N/A	N/A	N/A
Avahan– Project Parivartan	Blankenship 2008	No	No	No	No	No	NA	No	No
	Erausquin 2012	No	No	Yes	N/A	No	N/A	N/A	N/A
	Erausquin 2015	No	No	No	No	No	N/A	N/A	N/A
Empowerment for MSM	Zimmerman 1997	Yes	Yes	Yes	No	No	No	Yes	No
100% Condom Campaign	Kerrigan 2006	No	Yes	No	No	No	N/A	NR	NR
BIRDS	Halli 2006	No	Yes	No	No	Yes	N/A	NR	NR
Projeto Princesinha	Benzaken 2007	No	No	Yes	N/A	No	N/A	N/A	N/A
Fio da Alma	Kerrigan 2008	No	No	Yes	N/A	No	N/A	N/A	N/A
FPP	Gutierrez 2010	No	Yes	Yes	Yes	No	N/A	Yes	Yes
Encontros	Lippman 2010_a	No	Yes	No	No	No	N/A	NR	NR
	Lippman 2010_b	Yes	No	Yes	No	No	NR	N/A	N/A
	Lippman 2012	Yes	Yes	Yes	No	No	No	No	NR
Kapihan	Urada 2016	Yes	No	Yes	N/A	No	Yes	N/A	N/A
Shikimana	Kerrigan 2020	Yes	Yes	Yes	No	Yes	Yes	NR	NR
***Clubs***: *Forming peer groups for support and skills building*									
HIV prevention for married women	Tripiboon 2001	Yes	Yes	Yes	Yes	Yes	Yes	Yes	Yes
Microenterprise services among sex workers	Odek 2009	Yes	No	Yes	N/A	No	Yes	N/A	N/A
Peer network among methamphetamine users	Sherman 2009	Yes	Yes	Yes	Yes	No	Yes	No	Yes
AGEP	Austrian 2020	Yes	Yes	Yes	Yes	Yes	Yes	Yes	Yes
***Companionship***: *Expanding personal networks*									
Relational model for HIV prevention among drug users	Chen 2005	Yes	No	Yes	N/A	No	Yes	N/A	N/A
Gay bar-based Participatory Entertainment-Education	Gao 2007	Yes	Yes	Yes	No	No	Yes	Yes	NR
***Connections***: *Facilitating ties among non-peers*									
Youth-friendly sexual and reproductive health intervention	Aninanya 2015	Yes	Yes	Yes	Yes	Yes	No	No	NR
C2P Adaptation	Galai 2015	No	Yes	Yes	Yes	Yes	N/A	Yes	NR
***Cross-cutting interventions***: *Combining social mobilization with peer group formation*									
IMAGE	Kim 2009	Yes	Yes	No	Yes	Yes	Yes	Yes	NR
	Pronyk 2006	Yes	Yes	Yes	Yes	No	Yes	Yes	No
	Pronyk 2008	Yes	Yes	Yes	Yes	Yes	Yes	Yes	Yes
DREAMS	Gourlay 2019	Yes	Yes	Yes	No	Yes	No	NR	NR
	Gourlay 2022	Yes	Yes	Yes	No	Yes	No	NR	NR
SAPPH-IRe	Cowan 2018	No	Yes	No	Yes	No	N/A	Yes	N/A
Sisters with a Voice	Ndori-Mharadze 2018	No	No	Yes	N/A	No	N/A	N/A	N/A

*N/A * not applicable; *NR * not reported

## Summary of Studies by Category

Below we outline definitions of categories included in the typology and describe studies within each category.

### Social Mobilization (Collaboration)

Studies in this category sought to strengthen social ties within communities as part of community-wide mobilization campaigns. While these campaigns mostly did not form specific groups within a community, collaborating with community members or otherwise strengthening existing or forging new ties were key. All studies involved mobilization within general geographical areas (e.g., village or district).

Three studies, described below, were classified as social mobilization interventions. All studies utilized community mobilization as their theoretical basis, and all were cluster randomized trials (CRT).

The pair-matched CRT SASA! study in Uganda used social mobilization to reshape norms around IPV and shift power imbalances to reduce violence and HIV risk within communities. The SASA! approach encouraged strengthening connections among community members to build “power within” and support change within networks, among other intervention components. Findings included higher levels of condom use and HIV testing and fewer concurrent sexual partners among men. Effects among women trended in a similar direction but were smaller [39, 41].

The SHARE study in Uganda involved a multifaceted community mobilization intervention centered on advocacy, capacity strengthening, community activism, and information sharing. As part of community activism, intervention communities formed “IPV watch groups” and community action groups. Although the intervention involved advocacy efforts geared toward policy makers, it is unclear whether communities were directly involved. The study is noteworthy as it found significant reductions in HIV incidence as well as incidence of IPV (physical and sexual) across intervention and control groups [45].

The adapted “One Man Campaign” study in South Africa involved a theory-based community mobilization intervention to increase HIV testing in rural South Africa. The intervention aimed to impact six domains of community mobilization, including building shared concern around HIV prevention, creating critical consciousness, developing a well-connected organizational structure, cultivating individual and institutional, fostering collective actions, and social cohesion. Using an intent-to-treat analysis, the study found no significant changes in HIV testing behavior but found evidence that the hypothesized pathway to change (i.e., community mobilization) was associated with changes in HIV testing, as having higher village-level community mobilization scores were associated with increased odds of HIV testing [42, 46].

### Group Formation/Mobilization for Empowerment Among At-Risk Groups (Collectivization)

Studies in this category often had a purpose similar to social mobilization mentioned above, but here interventions focused on building ties within a specific, marginalized subpopulation, not just general community-wide mobilization. The aim was to help facilitate collection action and empowerment at the group and/or individual level (i.e., to help address upstream social determinants of health).

We categorized thirteen studies as collectivization. Most studies focused on collectivizing activities among sex workers (*n* = 11); one study focused on MSM [80] and another on both sex workers and MSM [78]. Most interventions took place in India, although there were exceptions [52–54, 76, 77, 80]. Interventions varied greatly, ranging from a single-session intervention to mobilize sex workers regarding human rights and HIV prevention [57] to programs spanning decades, such as the Sonagachi Project, a sex worker collective in existence for more than 30 years that continues to evolve [10, 50, 56]. Many interventions involved components that addressed both upstream and downstream factors related to risk, such as having sex workers serve as peer educators to increase awareness and build skills (to downstream factors) while simultaneously fostering collective action (to address upstream factors). Most interventions used quasi-experimental designs, such as serial cross-sectional designs, and most demonstrated success through significant changes in outcomes such as increased HIV-related knowledge, condom use, and decreases in sexually transmitted infections (STIs) [10, 47, 48, 50–52, 54, 56, 57, 77, 78, 80]. One study, the Fio da Alma study in Brazil, utilized a serial cross-sectional design and found null results but noted contextual challenges and low intervention participation as contributing factors [53]. This study sought to integrate community development activities into an existing HIV and STI peer education program, including creation of a Drop-In Center and encouraged participation in a newly formed sex worker organization. Two studies using more rigorous designs—group randomized trials—replicated the Sonagachi Project’s approach to sex worker empowerment within different parts of India [47, 56]. They found significant increases in HIV prevention behaviors, such as condom use [47], as well as increases in intermediate outcomes, such as increased social support [56]. A community randomized trial in Tanzania that utilized a community empowerment model and combination prevention approach found significant reductions in HIV incidence among female sex workers comparing intervention to control communities [76]. A cross-sectional study of another sex worker empowerment intervention in India, the Belgaum Integrated Rural Development Society (BIRDS) intervention, is noteworthy in that it developed a collectivization index based on membership in the local collective and contact with peer educators, and found that outcomes, like condom use and knowledge, were most improved among women with the highest collectivization scores [51].

The Avahan Initiative was a large-scale project across multiple states in India that worked to reduce HIV and STI risk among key populations—predominately female sex workers—through providing a combination package of services and strategies addressing both proximal and distal drivers of HIV [58–71]. Fostering empowerment and community mobilization were central components of the intervention. Fourteen studies included in the review evaluated Avahan [58–71], and three additional studies evaluated Project Parivartan, an intervention implemented under the Avahan umbrella in Andhra Pradesh [72–74]. Notably, one Avahan study focused on activities related to Durbar, a sex worker collective in Kolkata established by the Sonagachi Project (described above), as this intervention served as a model for scale-up within Avahan [62]. Many Avahan evaluations used serial cross-sectional data, or comparison of more vs. less-intense implementation across districts, to assess the relationship of program exposure and outcomes of interest, including condom use, with all finding some evidence of effectiveness [58, 61, 62, 65–70]. One Avahan evaluation used time trends from serial cross-sectional surveys and mathematical modeling to demonstrate that Avahan plausibly led to a reduction in HIV transmission among sex workers and their clients [60]. Other Avahan evaluations assessed pathways through which change occurred, including examining intermediate outcomes related to community mobilization and empowerment, either through propensity score matching [63, 71], structural equation modeling [64], or theoretically-informed secondary data analyses [59]. Results suggest that health-related outcomes are mediated through constructs relevant to social tie building/strengthening, such as collective action, collective agency, and individual self-efficacy. Several studies highlighted differential results across locations, socioeconomic status, and other factors highlighting the critical role of context, complexity, and historical programming on influencing intervention impact [62, 63]. Studies from Avahan’s Project Parivartan found overall similar results suggesting that program exposure was associated with increased condom use [73], and that intermediate outcomes related to empowerment played a key mediating role [72].

### Group Formation Among Peers and Personal Network Building (Clubs)

This type of intervention sought to create or strengthen peer groups, often through development of “clubs,” typically to facilitate peer-based support. These interventions often involved training on some sort of skill, such as income generation, or creating a physical space where groups could meet. Unlike collectivization interventions, “clubs” seldom mention collective action or advocacy, although several mention individual-level empowerment. The emphasis for clubs is mostly on individual downstream health-related factors (i.e., psychosocial support), not upstream factors (i.e., facilitating structural change).

Four studies were categorized in the “club” category, although many also involved community mobilization components. One study focused on forming support and/or skills-building groups for sex workers [55], one among youth [87], and one each among methamphetamine users in Thailand [82] and married women in rural Thailand [88].

The study among youth, the Adolescent Girls Empowerment Program (AGEP) in Zambia, aimed to build both short term social, health, and economic assets for adolescent girls, as well as to achieve long-term changes in sexual behavior, early marriage, and education [87]. The two-year intervention involved a weekly girl group meeting facilitated by a trained peer (i.e.), young woman, provision of health vouchers to girls for sexual and reproductive health (SRH) services, and opening of adolescent-friendly savings account [87]. Through a cluster randomized trial involving four provinces, the study found “modest, positive” impacts on SRH knowledge, financial literacy, savings behavior, self-efficacy, and transactional sex within four years of implementation [87]. However, the study found no effect on primary education, fertility outcomes, gender equity norms, HIV knowledge, or acceptability of gender-based violence, suggesting intervention activities fell short of building a comprehensive set of assets to alter longer term behaviors and outcomes [87].

Several studies integrated peer activities related to micro-enterprises, savings, or other means of financial empowerment, as well as support and education, among both sex workers [55] and adolescents [87]. One before/after micro-enterprise study in Kenya among sex workers demonstrated improved outcomes related to consistent condom use and reductions in numbers of weekly sexual partners [55]. A group randomized trial in Thailand among young adults who use methamphetamine found that both the micro-enterprise intervention group and life-skill control group demonstrated similar reductions in methamphetamine use and increases in consistent condom use [82].

The final intervention sought to improve ways married women in rural Thailand could prevent HIV, such as through improving condom negotiation skills with their husbands [88]. The intervention, grounded in the community development model, sought to mobilize communities through establishing informal, village-based organizations and networks for HIV prevention and delivering a 3-session program to married women facilitated by a trained health volunteer [88]. Authors noted that women received social support from other women during these sessions [88]. The study utilized a CRT design among eight communities (four assigned to the intervention), and found that married women in intervention communities reported more condom use and condom negotiation skills compared to control communities [88].

### Expanding Personal Networks (Companionship)

Interventions in this category focused on building personal networks of specific individuals, without formation of a group. The purpose of network expansion could be to find peers sharing similar characteristics to increase access to information and reduce stigma (e.g., expanding personal networks of MSM to increase access to information on condom use, HIV testing, etc.) or, conversely, to expand an individual’s network to others who do not share specific characteristics (e.g., expanding personal networks of people who inject drugs to include people who do not inject drugs).

Two small studies conducted in China evaluated interventions that intentionally sought to expand personal networks of individuals belonging to specific populations [79, 81]. In the study involving people who inject drugs [81], a prevention worker provided HIV prevention education and spoke to family members about how to support and help their family members build a supportive network of non-injecting friends. Results from the before/after pilot study, conducted among 100 women who inject drugs, found the intervention improved HIV knowledge, increased condom use, and decrease needle and syringe sharing [81]. Another study among MSM [79] involved a culturally centered participatory communication approach to promote safer sexual behavior, including through hosting activities encouraging people to expand their personal networks and empowering individuals to share new learnings with their peers. The study utilized a non-randomized pre/post design among 160 individuals, with half residing in areas where the intervention was not occurring (comparison group) and the other half residing within intervention areas. Results demonstrated substantial increases in knowledge and condom use comparing pre-to-post within the intervention group, with no such changes seen among the comparison [79].

### Facilitating Social Ties Among Non-peers (Connections)

Studies in this category relate to the concept of “bridging” social capital [89], whereby interventions attempted to bring together individuals from different levels of power, experiences, and roles to forge common understandings, innovation, and access to ideas and resources that would have been inaccessible otherwise.

Two studies focused on building ties with non-peers. One CRT in rural Thailand adapted the US-focused “Connect to Protect” program to bring together coalitions of diverse stakeholders to address structural factors impacting methamphetamine use and HIV risk behaviors among youth. Coalitions met regularly and included representatives from education, health, law enforcement, and local leaders. The study found no significant impact on methamphetamine use and HIV risk among young people but noted several implementation challenges that could have contributed to the null result, including civil unrest and active drug enforcement efforts in control communities [84].

Lastly, a study in Ghana examined an intervention aiming to increase youth’s access to sexual and reproductive services, including HIV prevention services, through providing a school-based curriculum, out-of-school outreach, community mobilization, and health worker training in youth-friendly services. The intervention encouraged dialogue and interaction between adolescents and providers and assessed barriers and facilitators to accessing services. Utilizing a CRT design, the study found that youth exposed to the intervention had significantly greater odds of using services compared to youth in control communities [83].

### Cross-Cutting Interventions

Four studies encompassed multiple types of social tie building interventions and have therefore been classified as “cross-cutting” interventions in the typology. All studies used both wider social mobilization strategies coupled with building peer groups to enact change.

The IMAGE study in South Africa included both intervention components related to social mobilization (“collaboration”) and building groups among peers (“clubs”). The study randomized both communities and individual women within intervention communities to receive a group-based microfinance and HIV education program, which represents the “club” component of the intervention as it aimed to build ties within peer groups. The study also identified women from the group program for additional training in leadership and mobilization, theorizing that because group-based learning can foster collective action, this momentum could be leveraged to engage women in wider community mobilization efforts around HIV prevention and intimate partner violence (IPV), which represents the social mobilization component of the intervention. The study found that rates of violence experienced by program participants was reduced by over 50% as compared to non-program participants but found no significant differences in outcomes, including HIV incidence, comparing intervention to control communities.

Another study involved the DREAMS (Determined, Resilient, Empowered, AIDS-free, Mentored and Safe) Partnership, which implemented a complex set of interventions to reduce HIV-related risk among adolescent girls and young women (AGYW) in South Africa and Uganda. The study evaluated the program through comparing outcomes by DREAMS exposure among randomly selected AGYW research cohorts in each location using quasi-experimental methods, including propensity score analysis [85, 86]. Notably, the study highlighted its use of “layering,” which was defined as receiving multiple interventions from a bundle of interventions implemented across socioecological levels. At the individual level, building “social assets” was one intervention that involved creating “safe spaces” where adolescent girls could go to socialize with other girls and gain information and mentorship [86]. At the contextual level, the intervention also sought to strengthen families and mobilize communities [86]. Therefore, this study also used both peer group formation and social mobilization to enact change. Using propensity score analyses, the study found that DREAMS consistently and positively impacted social support and less consistently impacted general self-efficacy [85]. The final two studies involved offering sex workers support groups for either pre-exposure prophylaxis (PrEP) or antiretroviral therapy (ART) (depending on HIV status) through an enhanced version of Sisters with a Voice—the SAPPH-Ire study [49]—a sex worker program implemented nationally in Zimbabwe, and coupling Sisters with a Voice with enhanced community mobilization [75]. Using a CRT design, the SAPPH-Ire study found that the intervention increased HIV testing and diagnosis among sex worker communities as compared to usual care communities [49]. The serial cross-sectional study that coupled Sisters with a Voice with intensified community mobilization similarly showed increased in service utilization across sites [75].

## Discussion

This review included 28 studies examining interventions that sought to build or strengthen social ties to impact HIV prevention outcomes in low- and middle-income countries. Within these studies, we identified five intervention types, including community-wide social mobilization (“collaboration”), formation of collectives to address both upstream and downstream factors related to health (“collectivization”), forming or strengthening groups to enhance peer support and build skills (“clubs”), expanding personal networks among individuals (“companionship”), and strengthening ties between heterogeneous groups/non-peers (“connections”). Most studies found that interventions were associated with positive health-related changes, such as reduced HIV incidence, increased condom use and other HIV prevention-related behaviors, and increased health service utilization. However, some interventions fell short of their stated goals, such as the IMAGE study [40, 43, 44]and the Adolescent Girls Empowerment Program [87], showing only partial effectiveness or producing significant results only among certain subgroups. Other interventions, such as the One Man Campaign in South Africa, found null effects overall but evidence that the hypothesized pathways to change through social tie building generated some individual-level change [42]. Overall, these results suggest that efforts to build or strengthen social connections can effect change, although intervention complexity and context make it difficult to determine whether such interventions “work.” Additionally, teasing out whether interventions altered social ties directly was challenging given lack of measurement and inconsistent application of theory across studies. Below, we elaborate on these findings and suggest potential steps to further our understanding of how social ties can be built or strengthened to prevent HIV.

### Need for Theoretical Clarifications and More Measurement in Social Tie-Building Interventions

Although many interventions were grounded in theory, studies operationalized theories in different ways, i.e., what was defined as “mobilization” in one intervention was different from how it was defined in another. This finding is understandable in that many interventions were built from complex sociological theories that are themselves ambiguous or have multiple theoretical conceptualizations, as is the case with social capital [90]. It is partly this finding that led our team to categorize interventions by focusing not on theories used, but instead on what interventions did regarding deliberate attempts to strengthen or build social ties. Using theory is critical to designing sound social and behavioral interventions, and as part of grounding intervention design in theory, it is important to provide specificity around the function(s) of various intervention components and how they are hypothesized to effect change. Therefore, it is important that studies better specify intervention components, outline specific pathways through which the components are hypothesized to work, and measure whether change occurred. In this review, only 12 of 28 studies measured social tie building in some capacity. Interventions that intentionally measured social ties were better positioned to explain results and demonstrate pathways through which social tie building created change. One such example is the One Man Campaign in South Africa that found no overall impact of the intervention on community-wide HIV testing but did find evidence the intervention improved testing among those with direct exposure and who achieved higher mobilization scores using measures that were validated and theoretically-based [42].

### Social Relationships and Social Structures are Complex and Rooted in Context

Many interventions involved complex interventions to solve complex problems, often grounded in health inequities—problems spanning structural constraints, societal norms, and a host of other contextual realities. Several interventions attempted to impact social ties at multiple levels, or conversely, some sought to use social tie building to address both upstream and downstream factors simultaneously, such as through forming groups to mobilize and carry out collective action to address systemic inequities (upstream) while also leveraging group bonds to build social support and self-efficacy (downstream). In many complex interventions, striving to change upstream social and structural factors related to health, specifically those in the “collaboration” category that focused on community mobilization, results often fell short, especially as related to achieving long-term impact or diffusion of effects to those not directly involved in the intervention. These results may reflect the realities and limitations of attempting to address social and structural drivers of HIV through implementing a singular, albeit multifaceted, intervention, despite investigators’ best efforts to unpack these drivers and operationalize frameworks to address them. As highlighted by Auerbach and colleagues, these social drivers are “complex, fluid, non-linear, and contextual” and require situational and contextual characterization [91].

Context most likely played a major role in these interventions’ successes or failures, but as has been noted previously, investigators seem more likely to ascribe intervention failures to contextual challenges instead of crediting context as a facilitator for success [92]. One example of this from our review is that extensive work has been undertaken in India regarding sex worker collectivization and empowerment, mostly showing positive results, and to a lesser extent in other regions of the world, including Latin America and sub-Saharan Africa. Prior reviews have demonstrated the effectiveness of community empowerment interventions for HIV prevention among sex workers [93, 94]. This type of intervention has not been extensively implemented elsewhere, or with other key populations, likely due to context and challenges with mobilizing populations defined by certain behaviors or identities (e.g., MSM or people who inject drugs) versus occupation (e.g., sex work). Conversely, interventions falling into the “clubs” category covered wider geographical areas and populations, including AGYW, although notably this intervention type is mostly not attempting to address upstream factors, thus likely removing much of the complexity regarding outcomes and pathways to impact. Notably, we found few studies within the “companionship” and “connections” categories. This finding could be related to the complex connection between personal connections, social support, and HIV prevention [95], and challenges related to formulating such attempts in standardized interventions.

### Way Forward: Measuring Social Tie Building and Thinking Creatively About Study Design

Attention is needed to use study designs best suited to evaluate the effectiveness of complex interventions, especially those attempting to address upstream social and structural factors related to HIV vulnerability. More innovative thinking is also needed about how to define “effectiveness” for these types of interventions [91]. As an example from this review, the Avahan Initiative used a comprehensive evaluation design framework involving program monitoring, mathematical modeling, and cost effectiveness analyses [96]. Though not a rigorous study design in traditional terms, Avahan’s diverse and complementary evaluation strategies allowed investigators to delve into intermediate pathways along the casual model, including those involving social tie strengthening, and to recognize and understand the impact of context and complexity on differential intervention impacts. These analyses provided important lessons on implementing complex interventions in “real-world” settings, meaningful engagement of communities and diverse stakeholders, and challenges and sustainability of community-led processes [97]. Moving forward, thinking broadly about effectiveness and implementation for complex interventions seeking to build social ties to impact social and structural factors is needed.

### Strengths and Limitations

Despite a comprehensive search, it is possible eligible studies were missed. The definitional ambiguity of social ties could have contributed to missing relevant studies, or conversely, including studies that might not have been trying to intentionally alter social ties. This was often challenging to distinguish, especially for “club” interventions that sought to build ties within a group setting, often through skill-building activities. Frequently it was unclear whether the intervention was deliberately trying to build ties, or whether tie-building was simply a side effect of the group-based intervention format. Additionally, categories in the typology are not mutually exclusive, and often interventions attempted to intervene across different levels/types of social ties, such as the DREAMS intervention that sought to mobilize communities as well as strengthen ties among AGYW [85, 86]. If this typology becomes useful to other researchers and is used to define interventions, making further refinements will be important. Another limitation is that we synthesized results by study, not by specific intervention component and subsequent hypothesized mechanisms for achieving outcomes. Mostly this was because studies themselves did not provide this level of granularity, although the One Man Campaign [42] and the Avahan studies are notable exceptions [59, 65]. Additionally, this review focused on interventions seeking to explicitly improve health, which might have excluded interventions less focused on health but more generally focused on well-being and social connectedness. Regarding interventions, most occurred before the introduction and roll-out of PrEP, which is becoming an important aspect of HIV prevention as access and options increase. In the future, it will be important to understand how interventions seeking to build or strength social ties impact HIV prevention outcomes in the era of biomedical prevention.

## Conclusions

We identified 28 studies evaluating different approaches to build or strengthen social ties in low- and middle-income countries, categorized as collaboration, collectivization, clubs, companionship, and connections. Studies generally found a positive impact on HIV prevention outcomes, although the complexity and context within which interventions occurred likely shaped the diverse effects seen across settings. Nevertheless, results demonstrate the plausibility of intentionally altering social ties in ways that positively impact HIV prevention outcomes, including through upstream and downstream pathways. Future work can continue to expand theory—particularly describing functional roles of social tie building—and measurement of both processes (the creation of social ties) and outcomes.
